# The dyadic self‐care experience of stroke survivors and their caregivers: A qualitative descriptive study

**DOI:** 10.1111/hex.13838

**Published:** 2023-08-04

**Authors:** Wenna Wang, Gianluca Pucciarelli, Yongxia Mei, Zhenxiang Zhang, Ercole Vellone

**Affiliations:** ^1^ Academy of Medical Sciences Zhengzhou University Zhengzhou Henan China; ^2^ School of Nursing and Health Zhengzhou University Zhengzhou Henan China; ^3^ Department of Biomedicine and Prevention University of Rome “Tor Vergata” Rome Italy; ^4^ Department of Nursing and Obstetrics Wroclaw Medical University Wrocław Poland

**Keywords:** caregiver, dyadic, experience, family‐centred care, qualitative study, self‐care, stroke

## Abstract

**Background:**

Promoting self‐care is the core response strategy of the global health system to the burden of stroke. Although self‐care in stroke represents a dyadic phenomenon, the dyadic self‐care experience of stroke survivors and their caregivers is often overlooked in clinical practice.

**Objectives:**

The aim of this study was to explore the dyadic self‐care experience of stroke survivors and their caregivers.

**Design:**

A descriptive qualitative design was used to conduct the study.

**Results:**

The Consolidated Criteria for Reporting Qualitative Research was used for study reporting. A total of 21 stroke survivor–caregiver dyads were recruited for this study between May 2022 and September 2022. Data were collected through semistructured interviews and analyzed using thematic analysis. In this study, four themes were identified: (1) *poor relationship quality of the dyads*, (2) *dyadic incongruence in managing stroke*, (3) *a slow and tiring dyadic self‐care process* and (4) *happy cooperation in coping with dyadic self‐care*.

**Discussion and Conclusion:**

Healthcare professionals should give greater consideration to the contradictions and disparities that may arise between stroke survivors and caregivers during the self‐care process. It is crucial for them to provide personalized and tailored support and interventions that can assist these individuals in achieving a more optimal balance in their dyadic self‐care.

**Patient/Public Contribution:**

Patients were involved in the formulation of interview questions for this study. No members of the public were involved in this study.

## 1 INTRODUCTION

Worldwide, stroke is prevalent, costly and disabling for more than 80 million people.^1^ The total number of prevalent strokes, deaths and disability‐adjusted life years due to stroke has increased steadily since 1990, reaching 101 million prevalent stroke survivors.^2^, ^3^ Therefore, more efficient stroke prevention and management strategies are urgently needed to halt and eventually reverse the stroke pandemic, and promoting self‐care in stroke survivors should be a priority.^4^

Self‐care has been defined theoretically by Riegel et al. as ‘the process of maintaining health through health promotion practices and disease management in the field of chronic diseases, which includes three core dimensions of self‐care maintenance, self‐care monitoring and self‐care management’.^5^ Self‐care represents a fundamental and integral part of the treatment of patients with chronic diseases such as stroke. Indeed, as described by studies, self‐care could significantly improve the physical and mental health outcomes of stroke survivors.^6^, ^7^ For example, a systematic review demonstrated that compared with usual care, self‐care‐related programmes could significantly improve self‐efficacy and quality of life in stroke survivors.^8^

However, self‐care could have an impact not only on stroke survivors but also on their caregivers. Indeed, as described by the literature, authors observed that self‐care programmes for caregivers could improve their self‐efficacy and negative emotions, especially for those who become caregivers for the first time.^9^ Furthermore, the involvement of caregivers in the self‐care of stroke survivors could improve survivors' outcomes, such as enhanced medication intake, reduced negative emotion and decreased need for emergency department visits.^9^, ^10^, ^11^, ^12^ However, it may have many problems, such as role management conflict between stroke survivors and caregivers in daily activities, which is not conducive to the dyadic management of diseases.^13^

Promoting self‐care has gradually become the core coping strategy for the increasing burden of stroke.^1^, ^4^ However, based on research conducted over the past two decades, the current state of self‐care behaviours amongst stroke survivors after discharge is not optimistic.^14^, ^15^, ^16^ Therefore, clinicians and researchers face the challenge of understanding the complex process of stroke self‐care. In addition, as observed in other chronic diseases, self‐care can be considered a dyadic phenomenon.^17^, ^18^ Self‐care that involves both stroke survivors and their informal caregivers not only has an impact on the afflicted individual but is increasingly considered to have an impact on both the stroke survivors' and the caregivers' health.^19^, ^20^, ^21^

While previous research has identified areas for improving caregiver preparedness in stroke care, health policies often overlook the comprehensive aspects of self‐care in stroke survivors and the role of caregivers. It is essential for researchers to conduct relevant studies that examine the interaction between stroke survivors and their caregivers in the self‐care process. Moreover, developing targeted intervention programmes for self‐care is of utmost importance.^4^, ^8^ Although there have been studies on self‐care in the stroke population, qualitative research specifically focusing on the self‐care experience and perspectives of stroke survivor–caregiver dyads is lacking. Understanding the unique experiences of stroke survivors and their caregivers regarding self‐care is essential for healthcare professionals, as it can inform the development of targeted intervention programmes that support both stroke survivors' self‐care and the valuable contributions of caregivers to self‐care. Furthermore, this knowledge can contribute to the development of a comprehensive instrument that captures the various aspects of self‐care (self‐care maintenance, self‐care monitoring and self‐care management,) in stroke survivors. Hence, the primary objective of this study is to explore the dyadic self‐care experience of stroke survivors and their caregivers through a qualitative approach.

## 2 METHODS

### 2.1 Study design

This qualitative study constituted a component of a multicenter mixed‐methods study designed to investigate the dyadic interaction between self‐care amongst stroke survivors and the contribution of caregivers to stroke survivors' self‐care. A qualitative descriptive approach was chosen for this study.^22^ From a philosophical perspective, this research approach is closely aligned with constructionism and critical theories, utilizing interpretative and naturalistic methods. The use of this approach yields a concise summary in everyday language, aiding in the comprehension of a specific phenomenon by health science researchers across disciplines. In this study, the qualitative descriptive approach facilitated a straightforward process of understanding the experiences and perspectives of stroke survivors and their caregivers regarding dyadic self‐care. The Consolidated Criteria for Reporting Qualitative Research was used for study reporting.^23^

### 2.2 Participants and setting

Purposive sampling was used to recruit participants for this study. Stroke survivors and caregivers, enroled after discharge from three cities in central China, were asked to participate in the study. Hospital and community healthcare professionals referred participants to the research team, and eligibility screening was conducted by the research team. The inclusion criteria of stroke survivors in this study were (i) over 18 years of age, (ii) ability to understand Mandarin, (iii) living in the community not less than 3 months following stroke and (iv) sufficient cognitive and communication ability to provide informed consent and cooperate with the interviews. The inclusion criteria for stroke caregivers were that the caregiver should be a family caregiver who does not need to be paid and who is willing to participate in this study. The research participants were primarily recruited through the following methods: (i) recommendations from healthcare professionals in departments such as neurology and rehabilitation in hospitals; (ii) recommendations from staff members at community health centres and (iii) referrals from the enroled research participants to recruit their acquaintances who were also stroke survivors.

### 2.3 Data collection

Before the interviews, the first author explained the purpose of the interviews to the participants through phone calls or face‐to‐face meetings, obtained their informed consent and scheduled interview times. Based on the participants' preferences, the interviews were conducted at the participants' homes, within the community, at community health service centres, rehabilitation centres or in parks. To get more information, the first author held separate semistructured individual interviews that were conducted with stroke survivor–caregiver dyads. In cases where there were inconsistent responses, joint interviews were conducted with both survivors and caregivers to obtain more accurate information. Stroke survivors and caregivers were asked similar interview questions (Supporting Information: File 1). For example, while the patient's interview question was, ‘What specific experiences do you have during the self‐care process?’, the caregiver's interview question was ‘What specific experiences do you have while participating in the patient's self‐care process?’. The first author who conducted the interviews is a nursing PhD student with practical internship experience in working with stroke survivors and caregivers. She has a lively and talkative personality and possesses the ability to conduct qualitative research. Data saturation determined the sample size, that is, interviews stopped only when no new data appeared within three consecutive interviews after analysis of at least 10 interviews.^24^ The interviews lasted an average of 65 min. The authors kept all the data such as audio recordings, transcripts, handwritten notes and consent forms in a locked drawer and their password‐protected computer files.

### 2.4 Data analysis

Data analysis was conducted using thematic analysis because, as a flexible and useful research tool, it provides a rich and detailed yet complex account of the data.^25^, ^26^ Forty‐two interviews (21 dyads interviews) of the participants were conducted and included in the analysis of this study. All the audio recordings of the interviews were transcribed verbatim by the first author, and the second author double‐checked the transcripts. The transcripts were read and reread to become immersed in the data, and notes were written to become familiarized with the data. Several meetings were held amongst the authors to discuss and reach an agreement on the coding. An initial thematic map was created based on the coding to form themes.

### 2.5 Rigour

This study ensured rigour through several strategies including credibility, dependability, confirmability and transferability.^27^, ^28^ Credibility was enhanced by (i) a semistructured interview guide summary based on the literature (Supporting Information: File 1), (ii) expertise and rich experience of all the authors in qualitative and stroke‐related research and (iii) emphasis on the importance of honest responses to all the questions and explanations. Dependability was primarily achieved by providing clear and detailed descriptions of the research process and ensuring consistency through discussions amongst team members during the data analysis process. Confirmability was achieved by including detailed descriptions using extracts of data from the findings. Finally, transferability was established by attempting variation sampling and providing a description as detailed as possible of the settings, dyads' characteristics, data collection and analysis process.

## 3 RESULTS

A total of 21 stroke survivor–caregiver dyads were interviewed to achieve thematic saturation between May 2022 and September 2022. The characteristics of the participants are presented in Table 1. Tables 2 and 3 provide more detailed information about stroke survivors and caregivers. Regarding stroke survivors, the mean age was 58.48 years. Sixteen (76.2%) were males, 9 (42.9%) had an education of high school diploma or higher and 18 (85.7%) were married. Thirteen (50%) of the 21 stroke survivors had ischaemic strokes, and more than 50% of survivors had a stroke one time. For caregivers, the mean age was 59.67 years, and most of them (80.9%) were females. Seventeen (80.9%) had an education or high school diploma or higher, and 18 (85.7%) were married. Seventeen (81%) of the 21 stroke caregivers were spouses, and 14 (67%) of them have chronic diseases.

**Table 1:** Sociodemographic and clinical characteristics of the stroke survivors and their caregivers.

Characteristics	Total (*N* = 42)	Survivors (*n* = 21)	Caregivers (*n* = 21)
Age, mean ± SD, y	59.07 ± 13.062	58.48 ± 13.411	59.67 ± 13.005
Sex, *n* (%)			
Male	21 (50.0)	16 (76.2)	5 (23.8)
Female	21 (50.0)	5 (23.8)	16 (76.2)
Level of education, *n* (%)			
High school diploma or higher	26 (61.9)	9 (42.9)	17 (80.9)
Diploma below high school	16 (38.1)	12 (57.1)	4 (19.1)
Marital status, *n* (%)			
Married	37 (88.1)	18 (85.7)	18 (85.7)
Single	2 (4.8)	1 (4.8)	1 (4.8)
Divorced/widowed	3 (7.1)	2 (9.5)	2 (9.5)
Employment status, *n* (%)			
Employed full‐time or part‐time	2 (4.8)	1 (4.8)	1 (4.8)
Unemployment or retired	40 (95.2)	20 (95.2)	20 (95.2)
Household's financial situation, *n* (%)			
After paying the bills, still have enough for special things that one wants	24 (57.2)	12 (57.2)	12 (57.2)
Have money to pay the bills, but only because one has to cut back on things	14 (33.3)	7 (33.3)	7 (33.3)
Have difficulty to pay the bills, no matter what one does	4 (9.5)	2 (9.5)	2 (9.5)
Diagnosis, *n* (%)			
IS		13 (61.9)	
HS		8 (38.1)	
Stroke event number, *n* (%)			
1		12 (57.1)	
2 or 3		6 (28.6)	
>3		3 (14.3)	
Course of the disease (y), *n* (%)			
<1		8 (38.1)	
1–3		4 (19.0)	
≥3		9 (42.9)	
ADL, *n* (%)			
Independence		11 (52.4)	
Minimal dependence		5 (23.8)	
Partial dependence		3 (14.3)	
Severe dependence		2 (9.5)	
Caregiver's relationship with survivors, *n* (%)			
Child			2 (9.5)
Spouse			17 (81.0)
Parent			2 (9.5)
Daily care time (h), *n* (%)			
<8			10 (47.6)
≥8			11 (52.4)
Healthy condition, *n* (%)			
No chronic diseases			14 (66.7)
Have chronic diseases			7 (33.3)

*Note*: The maximal score of the modified Barthel Index is 100 points, with scores of 80–100 representing independence, scores of 60–79 points representing minimal dependence, scores of 40–59 representing partial dependence and scores less than 40 denoting severe dependence.

Abbreviations: ADL, activity of daily life; HS, haemorrhage stroke; IS, ischaemic stroke.

**Table 2:** The detailed information of stroke survivors (*n*1 = 21).

Number	Age	Sex	Level of education	Marital status	Employment status	Household's financial situation	Diagnosis	Stroke event number	Course of the disease (month [M]/year [Y])	ADL
S1	63	Female	Diploma below high school	Married	Unemployment or retired	After paying the bills, still have enough for special things that one wants	IS	4	3 Y	Minimal dependence MBI = 75
S2	65	Male	High school diploma or higher	Married	Unemployment or retired	Have money to pay the bills, but only because one has to cut back on things	IS	1	10 Y	Independence MBI = 90
S3	70	Female	Diploma below high school	Married	Unemployment or retired	Have money to pay the bills, but only because one has to cut back on things	IS	1	6 Y	Independence MBI = 85
S4	33	Male	High school diploma or higher	Married	Unemployment or retired	After paying the bills, still have enough for special things that one wants	HS	1	1 Y to 6 M	Independence MBI = 85
S5	74	Male	Diploma below high school	Married	Unemployment or retired	After paying the bills, still have enough for special things that one wants	IS	2	8 Y	Partial dependence MBI = 50
S6	60	Male	High school diploma or higher	Married	Unemployment or retired	After paying the bills, still have enough for special things that one wants	IS	1	9 M	Partial dependence MBI = 55
S7	46	Male	Diploma below high school	Married	Unemployment or retired	After paying the bills, still have enough for special things that one wants	IS	1	5 M	Minimal dependence MBI = 60
S8	57	Male	Diploma below high school	Divorced	Unemployment or retired	Have money to pay the bills, but only because one has to cut back on things	HS	1	6 M	Minimal dependence MBI = 65
S9	32	Male	High school diploma or higher	Single	Unemployment or retired	Have money to pay the bills, but only because one has to cut back on things	HS	1	10 M	Severe dependence MBI = 35
S10	69	Female	Diploma below high school	Married	Unemployment or retired	After paying the bills, still have enough for special things that one wants	HS	4	4 Y	Partial dependence MBI = 50
S11	58	Male	High school diploma or higher	Married	Unemployment or retired	After paying the bills, still have enough for special things that one wants	HS	1	6 M	Independence MBI = 80
S12	68	Male	Diploma below high school	Married	Unemployment or retired	Have money to pay the bills, but only because one has to cut back on things	IS	3	20 Y	Independence MBI = 90
S13	51	Male	Diploma below high school	Married	Unemployment or retired	Have difficulty to pay the bills, no matter what one does	HS	2	9 Y	Independence MBI = 85
S14	66	Male	High school diploma or higher	Married	Unemployment or retired	Have money to pay the bills, but only because one has to cut back on things	IS	2	5 Y	Independence MBI = 80
S15	52	Male	High school diploma or higher	Married	Unemployment or retired	After paying the bills, still have enough for special things that one wants	HS	1	1 Y	Minimal dependence MBI = 75
S16	76	Female	Diploma below high school	Married	Unemployment or retired	After paying the bills, still have enough for special things that one wants	IS	6	2 Y	Independence MBI = 80
S17	61	Female	Diploma below high school	Married	Unemployment or retired	Have money to pay the bills, but only because one has to cut back on things	IS	2	1 Y	Independence MBI = 95
S18	51	Male	Diploma below high school	Married	Unemployment or retired	After paying the bills, still have enough for special things that one wants	IS	1	5 M	Minimal dependence MBI = 70
S19	59	Male	High school diploma or higher	Married	Employed full‐time or part‐time	After paying the bills, still have enough for special things that one wants	IS	1	4 M	Independence MBI = 85
S20	80	Male	Diploma below high school	Widowed	Unemployment or retired	After paying the bills, still have enough for special things that one wants	IS	2	3 Y	Independence MBI = 85
S21	37	Male	High school diploma or higher	Married	Unemployment or retired	Have difficulty to pay the bills, no matter what one does	HS	1	7 M	Severe dependence MBI = 30

Abbreviations: ADL, activity of daily life; HS, haemorrhage stroke; IS, ischaemic stroke; MBI, modified Barthel index.

**Table 3:** The detailed information of stroke caregivers (*n*2 = 21).

Number	Age	Sex	Level of education	Marital status	Employment status	Household's financial situation	Daily care time (h)	Length of care (months [M]/years [Y])	Caregiver's relationship with patient	Healthy condition
C1	65	Male	Diploma below high school	Married	Unemployment or retired	After paying the bills, still have enough for special things that one wants	<8	3 Y	Spouse	No chronic diseases
C2	64	Female	Diploma below high school	Married	Unemployment or retired	Have money to pay the bills, but only because one has to cut back on things	<8	10 Y	Spouse	Have chronic diseases
C3	71	Male	Diploma below high school	Married	Unemployment or retired	Have money to pay the bills, but only because one has to cut back on things	<8	6 Y	Spouse	No chronic diseases
C4	33	Female	High school diploma or higher	Married	Employed full‐time or part‐time	After paying the bills, still have enough for special things that one wants	<8	1 Y to 6 M	Spouse	No chronic diseases
C5	72	Female	Diploma below high school	Married	Unemployment or retired	After paying the bills, still have enough for special things that one wants	≥8	8 Y	Spouse	Have chronic diseases
C6	57	Female	Diploma below high school	Married	Unemployment or retired	After paying the bills, still have enough for special things that one wants	≥8	9 M	Spouse	No chronic diseases
C7	46	Female	Diploma below high school	Married	Unemployment or retired	After paying the bills, still have enough for special things that one wants	≥8	5 M	Spouse	No chronic diseases
C8	28	Male	High school diploma or higher	Single	Unemployment or retired	Have money to pay the bills, but only because one has to cut back on things	≥8	6 M	Child	No chronic diseases
C9	64	Female	Diploma below high school	Widowed	Unemployment or retired	Have money to pay the bills, but only because one has to cut back on things	≥8	1 Y	Parent	Have chronic diseases
C10	68	Male	Diploma below high school	Married	Unemployment or retired	After paying the bills, still have enough for special things that one wants	≥8	4 Y	Spouse	No chronic diseases
C11	56	Female	High school diploma or higher	Married	Unemployment or retired	After paying the bills, still have enough for special things that one wants	<8	6 Y	Spouse	No chronic diseases
C12	67	Female	Diploma below high school	Married	Unemployment or retired	Have money to pay the bills, but only because one has to cut back on things	<8	20 Y	Spouse	Have chronic diseases
C13	49	Female	Diploma below high school	Married	Unemployment or retired	Have difficulty to pay the bills, no matter what one does	<8	9 Y	Spouse	No chronic diseases
C14	66	Female	Diploma below high school	Married	Unemployment or retired	Have money to pay the bills, but only because one has to cut back on things	<8	5 Y	Spouse	No chronic diseases
C15	52	Female	High school diploma or higher	Married	Unemployment or retired	After paying the bills, still have enough for special things that one wants	≥8	1 Y	Spouse	No chronic diseases
C16	76	Female	Diploma below high school	Married	Unemployment or retired	After paying the bills, still have enough for special things that one wants	≥8	2 Y	Spouse	Have chronic diseases
C17	63	Male	Diploma below high school	Married	Unemployment or retired	After paying the bills, still have enough for special things that one wants	<8	1 Y	Spouse	No chronic diseases
C18	51	Female	Diploma below high school	Married	Unemployment or retired	After paying the bills, still have enough for special things that one wants	≥8	5 M	Spouse	No chronic diseases
C19	58	Female	Diploma below high school	Married	Unemployment or retired	After paying the bills, still have enough for special things that one wants	≥8	4 M	Spouse	No chronic diseases
C20	51	Female	Diploma below high school	Married	Unemployment or retired	After paying the bills, still have enough for special things that one wants	<8	3 Y	Child	Have chronic diseases
C21	69	Female	Diploma below high school	Widowed	Unemployment or retired	Have difficulty to pay the bills, no matter what one does	≥8	7 M	Parent	Have chronic diseases

**Figure 1:**
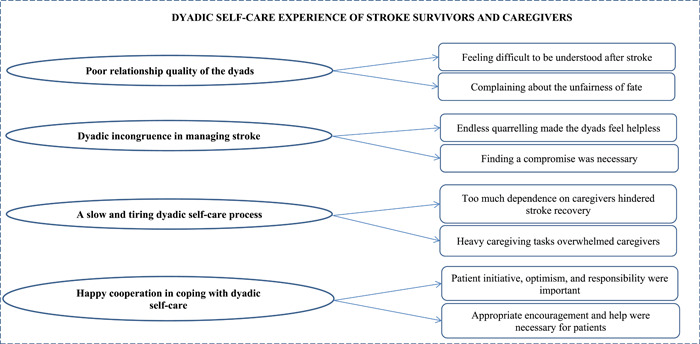
The themes and subthemes identified in this study.

The results of this study showed that, although caregiver involvement in the self‐care of stroke survivors provided critical support to stroke survivors, it was a complex process for the dyads to jointly participate in self‐care. From an overall perspective, although stroke survivor–caregiver dyads usually experienced various problems in the process of dyadic self‐care, it seems that good cooperation could help the dyads find new balance points to maintain the existing life after stroke. This study finally identified four main themes: (1) *poor relationship quality of the dyads*, (2) *dyadic incongruence in managing stroke*, (3) *a slow and tiring dyadic self‐care process* and (4) *happy cooperation in coping with dyadic self‐care*. The themes and subthemes identified in this study were illustrated in Figure 1.

### 3.1 Poor relationship quality of the dyads

As a major family event, stroke had a negative impact on the relationship between stroke survivors and caregivers.

#### 3.1.1 Feeling difficult to be understood after a stroke

The stroke survivors reported difficulties in obtaining understanding from others, both for themselves and caregivers. Additionally, several stroke survivors described their disappointment and powerlessness in dealing with the relationship with the caregivers after a stroke.

After falling ill, my emotional state became very unstable. I frequently argued with my wife, and our relationship reached the brink of divorce. I feel like the world has been cruel to me. Being disabled now, it seems that no one cares about me anymore. (S7)

He always complains that the food I prepare is too salty, yet he never bothers to cook himself. I don't want to say anything negative about him. I keep all my feelings bottled up inside (tears welling up uncontrollably)… I'll be fine… (S17)

Sometimes, instead of going out to exercise with me, she would stay at home and play with her cell phone. It made me feel very sad and hopeless about our future. (S13)

#### 3.1.2 Complaining about the unfairness of fate

Some stroke caregivers described their situation as feeling helpless, watching their normal lives slip away after the stroke. They expressed frustration with the high demands placed on them by the stroke survivors and even attributed their own misfortunes to the stroke. As a result, these caregivers had limited communication with the stroke survivors, which affected the quality of their relationship within the dyad and hindered their involvement in the self‐care of the stroke survivors.

For a long time, I struggled to sleep well at night. Whenever he woke up during the night, he would call me to help him turn over or move his arms and legs. There were times when I felt overwhelmed and couldn't bear it any longer. (C6)

Since her blood pressure is not very high (around 130/90 mmHg), I no longer remind her to take her medication. And we seldom communicate with each other at home. Anyway, I don't want to stay at home with her all the time. (C3)

### 3.2 Dyadic incongruence in managing stroke

This study found great differences in the attitudes and views of some stroke survivors and caregivers towards the secondary prevention of stroke, symptom or sign monitoring, and rehabilitation.

#### 3.2.1 Endless quarrelling made the dyads feel helpless

It was difficult for the dyads to come to an agreement, and communication conflicts easily occurred, which hindered them from participating in the dyadic self‐care. In addition, the disagreement relating to stroke management may contribute to anxiety and stress within the dyads.

Almost every day, we would argue about his smoking and drinking habits. He would defend himself, saying, ‘Drinking has nothing to do with stroke. Just look at Mao Zedong, he smoked excessively but lived until 83 years old’. I would urge him to cooperate with blood pressure measurements and exercise, but he believed they were futile. Our constant quarrels took a toll on my own mood, and I found myself becoming increasingly irritable. It pains me to admit all of this. (C5)

I consider myself fortunate to have encountered such a compassionate individual in my life. However, I find it challenging to endure the pain of living in this world. The difficulties I face make life too arduous, and I constantly feel overwhelmed by stress. (S10)

While stroke survivors acknowledged the importance of caregiver assistance in helping them navigate life transitions, they struggled to effectively manage conflicts and disagreements with their caregivers. Despite the passage of time, some individuals continued to grapple with the challenges arising from managing their stroke condition, resulting in ongoing dyadic incongruence.

Yesterday, I had a plan to complete 500 arm lifts and 200 leg lifts, but I was too exhausted to finish it. When she found out, she perceived me as being overly ambitious and insincere about completing the plan. Later, she insisted that I couldn't have dinner until I completed the exercises. I felt trapped and powerless, as if I were a prisoner and she was my captor. (S19)

Deep down, I felt grateful for his kindness towards me, especially during the numerous times I had a stroke. However, we had divergent perspectives when it came to illness, and he struggled to comprehend my thoughts. As a result, there were occasions when I would decline to exercise with him. (S16)

#### 3.2.2 Finding a compromise was necessary

The dyads described that when their views and opinions were different if they found a way to resolve them or one of them was willing to compromise, the contradiction between them about stroke self‐care could be resolved.

I asked him to go out for exercise. He felt that his walking appearance was too ugly to go out. I failed to persuade him many times, so I prepared rehabilitation equipment for him at home. (C20)

My mother and I consistently held different viewpoints regarding stroke prevention and rehabilitation. Whenever conflicts arose, we would seek the guidance of a doctor to help us find a resolution. Ultimately, we both placed trust in the words of the medical professionals. (S21)

### 3.3 A slow and tiring dyadic self‐care process

Stroke survivors often have symptoms such as inactivity, sleepiness and fatigue, which hinder their self‐care. This study found that stroke has brought great changes to the daily lives of stroke survivors and caregivers.

#### 3.3.1 Too much dependence on caregivers hindered stroke recovery

Some stroke survivors relied too much on the support of caregivers and became more and more passive or slothful in the process of treatment adherence and health‐promoting practices, behaviour and condition monitoring and managing signs and symptoms when they occur health behaviour maintenance.

Indeed, I was able to independently brush my teeth and wash my face, but due to the lack of strength in my arm, I faced challenges in performing various daily tasks. It became quite inconvenient for me to handle things on my own. I relied on my wife to assist me with washing my face, bringing me food, and administering medication… (S18)

For instance, I would encourage him to sit up and perform the hand‐raising gesture together as part of the Bobath therapy. However, he would often complain of fatigue and claim that sitting up was too tiring for him. When I pushed him to try, he would reluctantly perform a few repetitions, but as soon as I stopped urging him, he would immediately cease the activity. It seemed like he was only going through the motions to appease me. To make matters worse, his relatives and friends constantly remarked on his increasing weight gain. I felt helpless and unsure of what to do in such a situation. (C18)

#### 3.3.2 Heavy caregiving tasks overwhelmed caregivers

Upon assuming the role of a caregiver for a stroke survivor, individuals often found themselves burdened with additional daily household responsibilities. Many caregivers, particularly women, expressed a selfless dedication to caring for the stroke survivor. However, the overwhelming responsibility of caregiving took a toll on their well‐being, resulting in a decline in their physical and mental health. Caregivers reported dedicating nearly all their time to the needs of the stroke survivor, leaving them constantly fatigued and devoid of any energy or time for other pursuits.

Frankly speaking, this disease (stroke) was a suffering thing for him (stroke survivor) and me. I ate irregularly since my husband had a stroke. Alas, I felt very tired every day. Sometimes I put my meals on the table and fell asleep as soon as I lay on the sofa. And I found that my meals had not been eaten when I woke up. He exhibited significant laziness (due to muscle weakness and fatigue following the stroke), to the extent that I had to assist him and consistently encourage him to take care of himself. (C11)

This study found that the laziness of stroke survivors made the caregivers feel powerless and undertake more care tasks, while the caregivers' excessive contribution to self‐care of the stroke survivors made the survivors passive, which was not conducive to cultivating the stroke survivors' independence and autonomy in self‐care.

Sometimes I said to myself, ‘Let him do what he likes. And I shouldn't be angry with him because it's useless.’ Yeah, I had to care for him because he was a poor stroke survivor. (C15)

I believed I could walk independently for an extended period, but my mother constantly worried about the risk of falls. She would always assist me or insist on pushing me in a wheelchair. Instead of engaging in constant arguments, I eventually acquiesced and started listening to her concerns after several discussions. (S9)

### 3.4 Happy cooperation in coping with dyadic self‐care

As stroke survivors may have different degrees of dysfunction, such as limb dysfunction, they cannot be separated from the help and support of nursing staff after discharge.

#### 3.4.1 Patient initiative, optimism and responsibility were important

Some stroke survivors acknowledged that caregivers' assistance was not always available as caregivers also had their own lives to attend to. These survivors emphasized the need to take responsibility for their own health, collaborate with caregivers in self‐care efforts, and strive to establish a balance between the caregivers' role and their own independence. Consequently, these individuals demonstrated a proactive approach to self‐care, adapting to their post‐illness condition. This proactive approach primarily involved maintaining healthy behaviours, adhering to medication schedules and engaging in rehabilitation exercises as advised by their healthcare providers.

…I prioritize my health now! Since my stroke, I made significant lifestyle changes by quitting smoking and drinking. To ensure I take my medication on time, I always request my wife to organize the next day's doses in three small boxes and place them on the dinner table the night before. Having experienced a stroke previously, I am more vigilant and alert to any recurring symptoms. Given my current limitations with my leg and arm, I exercise extra caution when going out. Naturally, there are certain tasks I still rely on my wife to assist me with, as they are beyond my current capabilities. (S14)

Other stroke survivors mentioned that they should frankly accept the disease (stroke) and treat the changes in their own life, work, and family relations caused by stroke with an optimistic attitude. They believed that targeted and planned activities, positive emotions and the cooperation of their families were the key elements for successful self‐care.

I hesitated to ask for help repeatedly as it was challenging for me. There were instances when I would request a glass of water from my family, and they would respond with ‘wait a moment’. However, I often lost track of time, uncertain if it had been 10 or 15 min. Therefore, my primary objective became self‐care, ensuring that I could take care of myself to the best of my abilities. (S12)

I need her, but without her I could do more… I've always dealt with mobility limitations on my own, like I bought an electric wheelchair. I support her hobbies very much, and her optimistic attitude also makes me confident in life. (S2)

#### 3.4.2 Appropriate encouragement and help were necessary for the patients

The stroke caregivers described that it is important for stroke survivors to actively participate in self‐care after a stroke. And the role of caregivers was to help stroke survivors improve their self‐efficacy and provide emotional support. Some caregivers said that it was gratifying to see stroke survivors struggling with recovery, which reduced complaints and stress about their lives and allowed them to rebuild their lives.

I felt sorry for him when I saw how difficult it was for him to dress himself, but I knew that stroke survivors should not let others help them too much. He was very stressed and irritable after his illness, but I always tolerated and encouraged him and filled him with confidence and hope for the future. In addition, I also helped him make or buy some auxiliary exercise equipment to give him a safe and comfortable exercise environment. (C4)

I am a very cruel person, so I always forced my father to participate in exercise and made him responsible for his health. I would help him only when he really couldn't do it. (C8)

After being discharged from the hospital, I found myself confined to the house, constantly circling around her for nearly 24 h. The monotonous routine led to boredom and even sparked quarrels between us. Eventually, she started suggesting that we go to the park for a walk, allowing her to engage in some exercises. As a result, we both concluded that this change of scenery was beneficial for our overall well‐being. (C1)

He was always willing to take the responsibility of self‐care, and I was happy to take some of the care responsibility for him, too. I was responsible for cooking, helped him bathe… The two of us had a clear division of labour. After each of us had done our own things, we could spend our time as we please. (C12)

## 4 DISCUSSION

This study demonstrated that it was a complex process for the stroke survivor–caregiver dyads to jointly participate in self‐care after stroke. (1) *Poor relationship quality of the dyads*, (2) *dyadic incongruence in managing stroke*, (3) *a slow and tiring dyadic self‐care process* and (4) *happy cooperation in coping with dyadic self‐care* were identified as the main themes, which illustrated what the dyads experienced after discharge. Usually, poor relationship quality of the survivor–caregiver dyads after stroke was integral to a negative experience of dyadic self‐care, and dyadic incongruence in managing stroke made self‐care difficult. Passive or slothful stroke survivors and excessively contributing caregivers usually experienced a slow and tiring dyadic self‐care process, while positive or active stroke survivors and supportive caregivers were more likely to achieve happy cooperation in coping with dyadic self‐care.

Stroke, in which self‐care is particularly complex and involves treatment adherence and health‐promoting practices (self‐care maintenance), behaviour and condition monitoring (self‐care monitoring) and managing signs and symptoms when they occur (self‐care management), is a context or situation that places responsibility on both the stroke survivors and caregivers.^4^ However, in many countries, it is common for family members to care for stroke survivors after discharge from the hospital.^19^, ^29^ This study showed that a poor relationship within the dyads made them engage in self‐care with negative emotions. It has been proven that high mutuality has a positive impact on patient and caregiver outcomes.^30^ Future studies could promote the dyads to actively participate in self‐care by improving the mutual relationship between stroke survivors and caregivers.

For most stroke survivors and families, self‐care was associated with comanagement poststroke, which may cause contradictions and conflicts in the dyads.^31^ The phenomenon of disagreement between stroke survivors and caregivers in the process of self‐care is called dyadic incongruence.^18^ Participants in this study agreed that dyadic incongruence in managing stroke between stroke survivors and caregivers made them treat self‐care with a negative attitude and even leads to the reverse effect of caregivers' contribution to the self‐care of stroke survivors. In addition, disagreement between stroke survivors and caregivers about the arrangement for self‐care could be influenced by the less‐than‐optimal quality of the relationship between caregivers and stroke survivors, and this hypothesis needs to be verified in further studies. Studies have found that dyadic relationship communication is an important skill to promote dyadic disease management.^32^, ^33^ Therefore, it is important to identify the entry point and components of the most effective intervention and develop a tailored dyadic stroke self‐care programme to improve health outcomes for stroke survivors and caregivers with a Chinese background.

This study also revealed that conflicts arose when passive stroke survivors relied heavily on caregivers to assume more responsibilities in the self‐care process. Overdependence on caregivers can hinder stroke survivors from taking the initiative in their own self‐care. Additionally, previous studies have indicated that family support can have both positive and negative effects on the self‐care of stroke survivors.^13^, ^34^ That is to say, stroke survivors could not live without the support of caregivers, but excessive caregiver contributions will hinder stroke survivors from participating in self‐care and bring a greater burden to caregivers.

In addition, the participants in this study reported another dyadic self‐care type where active stroke survivors and supporting caregivers were more likely to achieve happy cooperation in self‐care. Active stroke survivors have higher self‐efficacy for rehabilitation, which is more conducive to the management of their own diseases.^14^ Based on these findings, healthcare professionals should adopt a holistic approach and consider stroke survivors and their caregivers as a unified unit in providing care. Customized educational interventions should be designed for each dyadic self‐care type to enhance effective disease management. Furthermore, it is essential to conduct longitudinal research to explore the stability of dyadic self‐care types over time and identify factors that influence their stability.

### 4.1 Limitations

This study has several limitations. Firstly, although the authors interviewed the stroke survivor and caregiver separately as much as possible, nearly half of the dyads (*n* = 9) participated in the interview at the same time, which might have prevented them from speaking freely and increased the risk of obtaining embellished information. Second, although this study included patients with varying disease durations, it did not demonstrate the influence of time on the dyadic self‐care experience of stroke patients and caregivers. Future longitudinal research could be conducted to further elucidate the evolving process of dyadic self‐care in patients and caregivers, leading to more scientifically comprehensive conclusions. In addition, China's unique traditional culture, such as ‘domestic disgrace should not be publicised’, may have hindered some participants from truly describing the contradictions and conflicts encountered in self‐care to a certain extent. Moreover, the sample, which was recruited from three cities in Henan Province, was not ethnically diverse, so further studies are required to assess the appropriateness of the findings for cross‐cultural transferability in China.

### 4.2 Implications for clinical practice and research

This study has several clinical and scientific implications. First, this study verified that self‐care for stroke is a complex dyadic process, and more targeted strategies need to be developed to empower and encourage individuals with stroke and their caregivers to engage in self‐care based on their real experiences. Second, the future stroke self‐care programme should focus on poor relationships and dyadic incongruence of the stroke survivor–caregiver dyads and take targeted measures to promote dyadic communication and cooperation. In addition, knowing the lived experiences of stroke survivors and caregivers regarding dyadic self‐care could help in developing a specific instrument that could analyze dyadic self‐care in stroke survivors and the contribution of their caregivers.

## 5 CONCLUSIONS

This study reaffirmed the significant role of stroke caregivers in the self‐care of stroke survivors, while also acknowledging the challenges they face in effectively managing the disease together. Recognizing the complexity of the dyadic disease management process, healthcare professionals should prioritize understanding the conflicts and disparities that arise between stroke survivors and caregivers during self‐care. By providing personalized and tailored support and interventions, healthcare professionals can assist in finding a better equilibrium in dyadic self‐care and promote a more harmonious and effective caregiving dynamic.

## AUTHOR CONTRIBUTIONS

Wenna Wang and Zhenxiang Zhang conceptualized and designed the study. Wenna Wang and Yongxia Mei developed the interview guide. Wenna Wang collected the data and transcribed the interviews. Wenna Wang, Gianluca Pucciarelli and Ercole Vellone analyzed and interpreted the data. Wenna Wang and Yongxia Mei wrote the first draft of the manuscript, Gianluca Pucciarelli and Ercole Vellone revised the manuscript critically for important intellectual content and Zhenxiang Zhang performed the quality control of this study. All authors have given final approval of the version to be published and agreed to be accountable for all aspects of the work in ensuring that questions related to the accuracy or integrity of any part of the work are appropriately investigated and resolved.

## CONFLICT OF INTEREST STATEMENT

The authors declare no conflict of interest.

## ETHICS STATEMENT

This study was approved by the Research Ethics Committee of Zhengzhou University (ZZUIRB2021‐115). All participants consented to the study verbally and in writing. Only the research team members had access to the audio tapes and transcripts of the original interviews.

## Supporting information

Supporting information.

## Data Availability

The data that support the findings of this study are available on request from the corresponding author. The data are not publicly available due to privacy or ethical restrictions.

## References

[1] Duncan PW , Bushnell C , Sissine M , et al. Comprehensive stroke care and outcomes: time for a paradigm shift. Stroke. 2021;52(1):385‐393. 10.1161/STROKEAHA.120.029678 33349012

[2] Feigin VL , Krishnamurthi RV , Parmar P , et al. Update on the global burden of ischemic and hemorrhagic stroke in 1990‐2013: the GBD 2013 study. Neuroepidemiology. 2015;45(3):161‐176. 10.1159/000441085 26505981 PMC4633282

[3] Roth GA , Mensah GA , Johnson CO , et al. Global burden of cardiovascular diseases and risk factors, 1990‐2019: update from the GBD 2019 study. J Am Coll Cardiol. 2020;76(25):2982‐3021. 10.1016/j.jacc.2020.11.010 33309175 PMC7755038

[4] Riegel B , Moser DK , Buck HG , et al. Self‐care for the prevention and management of cardiovascular disease and stroke: a scientific statement for healthcare professionals from the American Heart Association. J Am Heart Assoc. 2017;6(9):e006997. 10.1161/JAHA.117.006997 28860232 PMC5634314

[5] Riegel B , Jaarsma T , Strömberg A . A middle‐range theory of self‐care of chronic illness. ANS Adv Nurs Sci. 2012;35(3):194‐204. 10.1097/ANS.0b013e318261b1ba 22739426

[6] Boger EJ , Demain SH , Latter SM . Stroke self‐management: a focus group study to identify the factors influencing self‐management following stroke. Int J Nurs Stud. 2015;52(1):175‐187. 10.1016/j.ijnurstu.2014.05.006 24917370

[7] Ruksakulpiwat S , Zhou W . Self‐management interventions for adults with stroke: a scoping review. Chronic Dis Transl Med. 2021;7(3):139‐148. 10.1016/j.cdtm.2021.03.001 34505014 PMC8413126

[8] Fryer CE , Luker JA , McDonnell MN , Hillier SL . Self management programmes for quality of life in people with stroke. Cochrane Database Syst Rev. 2016;2016:010442. 10.1002/14651858.CD010442.pub2 PMC6450423 27545611

[9] Tielemans N , Visser‐Meily J , Schepers V , et al. Effectiveness of the restore4stroke self‐management intervention “Plan Ahead!”: a randomized controlled trial in stroke patients and partners. J Rehabil Med. 2015;47(10):901‐909. 10.2340/16501977-2020 26424327

[10] Jamison J , Graffy J , Mullis R , Mant J , Sutton S . Barriers to medication adherence for the secondary prevention of stroke: a qualitative interview study in primary care. Br J Gen Pract. 2016;66(649):e568‐e576. 10.3399/bjgp16X685609 27215572 PMC4979933

[11] Vloothuis JDM , Mulder M , Nijland RHM , et al. Caregiver‐mediated exercises with e‐health support for early supported discharge after stroke (CARE4STROKE): a randomized controlled trial. PLoS One. 2019;14(4):e0214241. 10.1371/journal.pone.0214241 30958833 PMC6453481

[12] Roth DL , Sheehan OC , Huang J , et al. Medicare claims indicators of healthcare utilization differences after hospitalization for ischemic stroke: race, gender, and caregiving effects. Int J Stroke. 2016;11(8):928‐934. 10.1177/1747493016660095 27435204 PMC5518936

[13] Satink T , Josephsson S , Zajec J , Cup EHC , de Swart BJM , Nijhuis‐van der Sanden MWG . Negotiating role management through everyday activities: narratives in action of two stroke survivors and their spouses. Disabil Rehabil. 2016;38(24):2354‐2364. 10.3109/09638288.2015.1129442 26854923

[14] Frost Y , Weingarden H , Zeilig G , Nota A , Rand D . Self‐care self‐efficacy correlates with independence in basic activities of daily living in individuals with chronic stroke. J Stroke Cerebrovasc Dis. 2015;24(7):1649‐1655. 10.1016/j.jstrokecerebrovasdis.2015.03.054 25997978

[15] Jang DE , Shin JH . Self‐Care performance of middle‐aged stroke patients in Korea. Clin Nurs Res. 2019;28(3):263‐279. 10.1177/1054773817740670 29103311

[16] Ren XR , Wei YY , Su XN , et al. Correlation between self‐perceived burden and self‐management behavior in elderly stroke survivors: a longitudinal observational study. Medicine. 2020;99(44):e22862. 10.1097/MD.0000000000022862 33126330 PMC7598859

[17] De Maria M , Ausili D , Lorini S , Vellone E , Riegel B , Matarese M . Patient self‐care and caregiver contribution to patient self‐care of chronic conditions: what is dyadic and what it is not. Value Health. 2022;25:1165‐1173. 10.1016/j.jval.2022.01.007 35337754

[18] De Maria M , Ferro F , Ausili D , Buck HG , Vellone E , Matarese M . Characteristics of dyadic care types among patients living with multiple chronic conditions and their informal caregivers. J Adv Nurs. 2021;77(12):4768‐4781. 10.1111/jan.15033 34487558

[19] Haley WE , Marino VR , Sheehan OC , Rhodes JD , Kissela B , Roth DL . Stroke survivor and family caregiver reports of caregiver engagement in stroke care. Rehabil Nurs. 2019;44(6):302‐310. 10.1097/rnj.0000000000000100 31689247

[20] Moons P , Prikken S , Luyckx K . Chronic illness as a ‘family disease’: the need for appropriate scientific methods for dyadic research. Eur J Cardiovasc Nurs. 2020;19(2):98‐99. 10.1177/1474515120902376 32041429

[21] Wang W , Lin B , Mei Y , Zhang Z , Zhou B . Self‐care interventions in stroke survivor–caregiver dyads: a protocol for systematic review and meta‐analysis. BMJ Open. 2021;11(12):e051860. 10.1136/bmjopen-2021-051860

[22] Colorafi KJ , Evans B . Qualitative descriptive methods in health science research. Health Environ Res Des J. 2016;9(4):16‐25. 10.1177/1937586715614171 PMC7586301 26791375

[23] Tong A , Sainsbury P , Craig J . Consolidated Criteria for Reporting Qualitative Research (COREQ): a 32‐item checklist for interviews and focus groups. Int J Qual Health Care. 2007;19(6):349‐357. 10.1093/intqhc/mzm042 17872937

[24] Francis JJ , Johnston M , Robertson C , et al. What is an adequate sample size? Operationalising data saturation for theory‐based interview studies. Psychol Health. 2010;25(10):1229‐1245. 10.1080/08870440903194015 20204937

[25] Braun V , Clarke V . Using thematic analysis in psychology. Qual Res Psychol. 2006;3(2):77‐101. 10.1136/bmjopen-2016-011631

[26] Vaismoradi M , Turunen H , Bondas T . Content analysis and thematic analysis: implications for conducting a qualitative descriptive study. Nurs Health Sci. 2013;15(3):398‐405. 10.1111/nhs.12048 23480423

[27] Doyle L , McCabe C , Keogh B , Brady A , McCann M . An overview of the qualitative descriptive design within nursing research. J Res Nurs. 2020;25(5):443‐455. 10.1177/1744987119880234 34394658 PMC7932381

[28] Caelli K , Ray L , Mill J . ‘Clear as Mud’: toward greater clarity in generic qualitative research. Int J Qual Methods. 2003;2(2):1‐13. 10.1177/160940690300200201

[29] Qiu X , Sit JWH , Koo FK . The influence of Chinese culture on family caregivers of stroke survivors: a qualitative study. J Clin Nurs. 2018;27(1‐2):e309‐e319. 10.1111/jocn.13947 28677123

[30] Pucciarelli G , Buck HG , Barbaranelli C , et al. Psychometric characteristics of the mutuality scale in stroke patients and caregivers. Gerontologist. 2016;56(5):e89‐e98. 10.1093/geront/gnw083 27114475

[31] Satink T , Cup EHC , de Swart BJM , Nijhuis‐van der Sanden MWG . The perspectives of spouses of stroke survivors on self‐management—a focus group study. Disabil Rehabil. 2018;40(2):176‐184. 10.1080/09638288.2016.1247920 28110542

[32] Bakas T , McCarthy MJ , Miller EL . Systematic review of the evidence for stroke family caregiver and dyad interventions. Stroke. 2022;53(6):2093‐2102. 10.1161/STROKEAHA.121.034090 35264010 PMC9133104

[33] McCarthy MJ , Garcia YE , Dunn DJ , Lyons KS , Bakas T . Development and validation of a quality of relationship intervention for stroke survivor‐family caregiver dyads. Top Stroke Rehabil. 2020;27(4):305‐315. 10.1080/10749357.2019.1690823 31782683

[34] Sadler E , Wolfe CD , Jones F , McKevitt C . Exploring stroke survivors' and physiotherapists' views of self‐management after stroke: a qualitative study in the UK. BMJ Open. 2017;7(3):011631. 10.1136/bmjopen-2016-011631 PMC5353340 28283483

