# Supplementary material for: The dyadic self‐care experience of stroke survivors and their caregivers: A qualitative descriptive study
Source: Health Expect. 2023 Aug 4;26(6):2325–39. doi: 10.1111/hex.13838 (PMC10632628; doi:10.1111/hex.13838)
Supplement: Supplementary file 1 — Supporting information. [file HEX-26--s001.docx]

**Supplementary File 1. Interview guide summary**

Interview guide summary of stroke survivors

| 1. What changes did stroke bring to you and your caregiver? (Such as physical, emotional, daily life, dyadic relationship, etc.) |
| --- |
| 1. What specific experiences do you have during the self-care process? (Including self-care maintenance, self-care monitoring, and self-care management) |
| 1. What is your perspective on caregiver involvement in your self-care? |
| 1. What barriers have you encountered in engaging in self-care together with the caregiver? |

Interview guide summary of stroke caregivers

| 1. What changes did stroke bring to you and patients? (Such as physical, emotional, daily life, dyadic relationship, etc.) |
| --- |
| 1. What specific experiences do you have while participating in the patient's self-care process (Including self-care maintenance, self-care monitoring, and self-care management) |
| 1. How do you view your own participation in the patient's self-care? |
| 1. What barriers have you faced when engaging in self-care with the patient? |
